# SARS-CoV-2 Post Vaccinated Adverse Effects and Efficacy in the Egyptian Population

**DOI:** 10.3390/vaccines10010018

**Published:** 2021-12-24

**Authors:** Marwa O. Elgendy, Ahmed O. El-Gendy, Abdulaziz Ibrahim Alzarea, Sarah Mahmoud, Saad S. Alqahtani, Alzhraa M. Fahmy, Hesham R. El-Seedi, Ahmed M. Sayed, Ahmed D. Alatawi, Mohamed E. A. Abdelrahim, Abdullah S. Alanazi

**Affiliations:** 1 Department of Clinical Pharmacy, Teaching Hospital of Faculty of Medicine, Beni-Suef University, Beni-Suef 62513, Egypt; marwaosamaelgendy@yahoo.com; 2 Department of Clinical Pharmacy, Faculty of Pharmacy, Nahda University, Beni-Suef 62513, Egypt; 3 Department of Microbiology and Immunology, Faculty of Pharmacy, Beni-Suef University, Beni-Suef 62513, Egypt; ahmed.elgendy@pharm.bsu.edu.eg; 4 Clinical Pharmacy Department, College of Pharmacy, Jouf University, Sakaka 72341, Saudi Arabia; aizarea@ju.edu.sa (A.I.A.); adalatawi@ju.edu.sa (A.D.A.); 5 Department of Clinical and Chemical Pathology, Faculty of Medicine, Beni-Suef University, Beni-Suef 62513, Egypt; Sarah.kh_ah@yahoo.com; 6 Department of Clinical Pharmacy, Collage of Pharmacy, Jazan University, Jazan 82511, Saudi Arabia; ssalqahtani@jazanu.edu.sa; 7 Pharmacy Practice Research Unit, Department of Clinical Pharmacy, College of Pharmacy, Jazan University, Jazan 82511, Saudi Arabia; 8 Tropical Medicine and Infectious Diseases Department, Faculty of Medicine, Beni-Suef University, Beni-Suef 62513, Egypt; alzhraamohamed@med.bsu.edu.eg; 9 Pharmacognosy Group, Department of Pharmaceutical Biosciences, BMC, Uppsala University, SE 751 24 Uppsala, Sweden; hesham.el-seedi@farmbio.uu.se; 10 International Research Center for Food Nutrition and Safety, Jiangsu University, Zhenjiang 212013, China; 11 Department of Chemistry, Faculty of Science, Menoufia University, Shebin El-Kom 32512, Egypt; 12 Department of Pharmacognosy, Faculty of Pharmacy, Nahda University, Beni-Suef 62513, Egypt; ahmed.mohamed.sayed@nub.edu.eg; 13 Clinical Pharmacy Department, Faculty of Pharmacy, Beni-Suef University, Beni-Suef 62513, Egypt; 14 Health Sciences Research Unit, Jouf University, Sakaka 72341, Saudi Arabia

**Keywords:** Sinopharm vaccine, COVID-19, IgG, SARS-CoV-2, anti-spike, adverse effects

## Abstract

Vaccines are the solution to overcome SARS-CoV-2. This study aimed to determine the post-Sinopharm vaccine safety-profile and immunity through antibody titers. Data were collected using a structured questionnaire from Egyptian participants who received two doses of Sinopharm vaccine. Data were divided into three parts, the first and second parts were to detect participants’ post-first and second dose symptoms and practices, and the third for the results of IgG anti spike protein antibodies test and laboratory tests. Pain, redness, swelling at the injection site, headache, fatigue, and lethargy were the most common post-vaccine symptoms for both first and second doses. Most of the participants felt mild or no symptoms after vaccination. The symptoms started mostly during the first day post-vaccination and lasted for no more than two days. Forty-nine percent of the participants resulted in positive antibodies tests on day 18 post-vaccination. The average antibody level for vaccinated participants with past SARS-CoV-2 infection was much higher than that for non-past infected participants. These vaccines’ administration methods need to be reevaluated by changing the dose, dose interval, adding a third dose, or mixing it with other vaccines with different techniques to improve their protection rates. Further studies are required to validate this finding.

## 1. Introduction

COVID-19 is a pandemic disease and spreads rapidly by close contact, resulting in the phenomenon of cluster infection in hospitals and families. COVID-19 vaccination is the best way to control the spread of the disease [1].

There are various COVID-19 vaccines which are available now for use all over the world, including Pfizer BioNTech, AstraZeneca, Moderna, Johnson & Johnson, Sinopharm, Sinovac, Sputnik V, and COVAXIN [2].

Sinopharm vaccine, also known as BBIBP-CorV, is an inactivated coronavirus vaccine [1]. It acts by stimulating the immune system to produce antibodies against SARS-CoV-2. Antibodies are proteins that help to prevent infections and can provide protection against reinfection [3].

The Sinopharm vaccine is a killed inactivated coronavirus that can be injected into the arm without causing infection [1]. When the person takes the Sinopharm vaccine, B cells produce antibodies that stick to the invader spike protein and prevent the virus from entering cells [3].

The clinical trials on the Sinopharm vaccine showed that BBIBP-CorV could protect against SARS-CoV-2 infection but did not show how long that protection lasts. There are two types of antibodies: one against nucleocapsid or N protein found inside the virus, and the other is against the spike or S protein found on the virus’s surface, which the virus uses to enter our body cells [4]. A previous study showed that people who have been infected with SARS-CoV-2 have antibodies against the N protein and the S protein [5]. However, people who have been vaccinated against SARS-CoV-2 have antibodies against the S protein (not against the N protein), which are known as anti-S or anti-spike antibodies [5]. This antibody testing provides important information to those who have not been able to test for SARS-CoV-2with polymerase chain reaction (PCR) and helps them to confirm that the illness they experienced was indeed SARS-CoV-2. This antibody testing is now considered a tool to detect whether a coronavirus vaccine gives a sufficient immune response or not [6].

Then, to evaluate the vaccine effect, we can measure the level of antibodies against the SARS-CoV-2 in the vaccinated person’s blood by a quantitative IgG anti-spike protein antibodies test.

This study aims to determine the post-Sinopharm vaccine symptoms and evaluate their effectiveness by measuring the level of antibodies against the SARS-CoV-2 in the vaccinated person’s blood by a quantitative IgG anti-spike protein antibodies test.

## 2. Methods

### 2.1. The Study Design

A cross-sectional study using an online survey was conducted from April to June 2021 in Egypt.

### 2.2. Sampling Method

An online survey was conducted, and the participants who received two doses of the Sinopharm vaccine participated in the study. Of all the participants, 48 agreed to do an IgG anti-spike protein antibodies test by ELISA on day 18 after vaccination with the Sinopharm vaccine.

Inclusion criteria:

- Healthy people or those with well-controlled diseases.
- Adults over 18 years old.
- People who have received two doses of the Sinopharm vaccine at a 21-day interval.
- Exclusion criteria:
- People who have severe clinical conditions or are receiving immunosuppressive therapy.
- Less than 18 years old.
- People who received one dose of the Sinopharm vaccine.
- People who did not receive two doses of the Sinopharm vaccine at a 21-day interval.
- Pregnant women.

### 2.3. Data Collection

Data was collected using a structured online questionnaire. An online survey was used to collect the data from all over Egypt. The time required to fill out the questionnaire was 5 min. The questionnaire items were designed according to the information and instructions published by the FDA [6].

The questionnaire was divided into four parts:

1. Participants’ demographic data: age, gender, marital status, educational level, current place of residence (city or country), and job.
2. Post-first dose symptoms and practices.
3. Post-second dose symptoms and practices.
4. Results of IgG anti-spike protein antibodies test and laboratory tests at day 18 after vaccination with Sinopharm vaccine. The survey was conducted from April to June 2021 in Egypt.

### 2.4. IgG Anti Spike Protein Antibody Test

The findings of the IgG anti spike protein antibodies test and laboratory testing were obtained 18 days following the second dose of the vaccine. The test was carried out in accordance with a previously published immunoassay-based technique [3] which was designed to quantify IgG antibody titers in human serum or plasma. The test was carried out using the chemiluminescent sandwich concept. In this procedure, the serum or plasma sample was first subjected to SARS-CoV-2-specific recombinant antigens (Thermo Fisher Scientific, Hamburg, Germany, RP87680) linked to magnetic beads. The antigen–antibody complex was treated with an alkaline phosphatase-conjugated antibody (Thermo Fisher Scientific, Hamburg, A-10648) against human IgG to form a sandwich immune-complex after bound/free separation. After a second bound/free separation, a luminous substrate was added to the solution to allow for luminescence measurement. The chemiluminescence intensity was measured at 450 nm, within 17 min after adding the substrate. The reaction chamber was held at 42 °C throughout the experiment. The concentrations of the samples were then detected using the standard curve equation while accounting for each sample’s dilution.

## 3. Results

### 3.1. Demographic Data of the Participants

A total of 120 (102 female) subjects participated in the study. Most of the participants were in the age group of 18 to 35 years (47.5%). In addition, 74% of the participants were married, 77% were city dwellers, and 55% were health care workers. The demographic data of the participants are shown in Table 1

### 3.2. Symptoms and Practices Following the First Dose of the SINOPHARM Vaccine

Regarding the symptoms after receiving the first dose of vaccine, the majority of the participants (52.5%) suffered from pain, redness, or swelling in the injection site, and 50% suffered from fatigue and lethargy. Fifteen percent of all the participants suffered from headaches. Furthermore, 10% of those who received the vaccine experienced fever, muscle pain, and a runny nose. Furthermore, 7.5% suffered from joint pain, 5% felt dizzy, and 2.5% suffered from sore throats, coughs, allergies, rashes, decreased appetite, and inflammation of the nervous system (including numbness, tingling, and loss of sensation). On the other hand, 25% of the participants answered that they did not feel any side effects or post-vaccine symptoms. There were gap differences in the answers to the question, “To what extent do you rate the severity of these symptoms and complications?”. Forty-nine percent answered that the post-vaccine symptoms and complications were mild, 18% answered that the symptoms and complications were moderate, and 8% answered that the symptoms and complications were severe. The majority of the participants (67.5%) answered that the post-vaccine symptoms appeared after the first dose within 24 h of receiving the vaccine. In terms of the persistency of the adverse events, 22.5% of the participants answered that the symptoms that appeared after the first dose persisted for one day, 30% answered that the symptoms persisted for two days, and 22.5% answered that the symptoms persisted for more than two days. Regarding after-vaccine practices, 22.5% answered that they took pain relievers after taking the first dose of the vaccine, while 77.5% did not need to take any pain relievers. Symptoms and practices following the first dose of the Sinopharm vaccine are shown in Table 2.

### 3.3. Symptoms and Practices Following the Second Dose of the Sinopharm Vaccine

Most of the participants took the second dose of the vaccine three weeks after the first dose and answered that they were not infected with coronavirus between the first and second doses (or suffered from severe symptoms of coronavirus for more than two days after receiving the first dose). Regarding the symptoms after receiving the second dose of vaccine, some of the participants (12.5%) suffered from pain, redness, or swelling at the site of infection, and 45% suffered from fatigue and lethargy. Ten percent of all the participants suffered from headaches. A total of 7.5% suffered from runny noses. Five percent suffered from sore throats, allergies, and rashes. A total of 12.5% of the participants suffered from muscle pain after taking the second dose. Two-point-five percent of them felt dizzy and suffered from fever. Of the participants, 37.5% answered that they did not feel any side effects or post-vaccine symptoms. There were gap differences in the answers to the question, “To what extent do you rate the severity of these symptoms and complications after the second dose?”. A total of 32.5% answered that the post-vaccine symptoms and complications were mild, 22.5% answered that the symptoms and complications were moderate, and 7.5% answered that the symptoms and complications were severe. A large fraction of the participants (45%) answered that the post-vaccine symptoms appeared after the second dose on the first day after receiving the vaccine. A total of 22.5% of the participants answered that the symptoms that appeared after the second dose persisted for one day, 15% answered that the symptoms persisted for two days, and 25% answered that the symptoms persisted for more than two days. Regarding the after-vaccine practices, 20% answered that they took pain relievers after taking the second dose of the vaccine, but 80% did not need to take any pain relievers. Symptoms and practices following the second dose of the Sinopharm vaccine are shown in Table 3.

### 3.4. Results of IgG Anti Spike Protein Antibodies Test and Laboratory Tests at Day 18 after Vaccination with Sinopharm Vaccine

Among the participants who agreed to do an IgG anti spike protein antibodies test on day 18 after vaccination with the Sinopharm vaccine, 16% of them had previously been infected with coronavirus during the four months before vaccination. Fifty-one percent of total participants showed negative results (less than 1 AU/mL) for the IgG anti-spike protein antibodies test, and 49% showed positive results (more than 1 AU/mL) for this test. The average positive result of quantitative IgG anti spike protein antibodies tests for cases with a previous coronavirus infection during the four months before vaccination was 147 AU/mL. However, the average positive results of quantitative IgG anti spike protein antibodies test for the cases with no previous coronavirus infection before vaccination was 40 AU/mL. Regarding gender, the average of positive results of quantitative IgG anti spike protein antibodies tests for males was 83 AU/mL, and for females, it was 50 AU/mL. Regarding age, the average of positive results of quantitative IgG anti spike protein antibodies tests for the age group (18 to 40 years) was 72 AU/mL, for the age group (41 to 60 years) it was 56 AU/mL and for age group (>60 years) it was 29 AU/mL Regarding the laboratory parameters for the positive IgG anti spike protein antibodies cases: average platelets was 263 × 109/L, average hemoglobin was 12.6 g/L, average leucocytes was 7.9 × 109/L, average neutrophils was 53%, average lymphocytes was 42%, average Erythrocyte Sedimentation Rate (ESR) was 21 mm/h, average serum glutamate oxalate transaminase (SGOT) was 20 U/L, and average serum glutamate Pyruvate Transaminase (SGPT) was 20.5 U/L.18 after vaccination with the Sinopharm vaccine. The results of IgG anti spike protein antibodies test and laboratory tests at day 18 after vaccination with Sinopharm vaccines are shown in Table 4.

## 4. Discussion

The coronavirus infection is a pandemic disease which still threatens the world [7,8,9]. Vaccination of people is a solution to prevent the spread of the virus [10]. During the last year, many coronavirus vaccines have been developed. However, to be widely used among the population, the vaccine must be safe and effective [11].

At the beginning of the study, the total number of cases that were registered in Egypt with COVID-19 was 205,732, including 156,574 cases that were cured and 12,210 deaths. (Available online: https://egcovac.mohp.gov.eg/#/home; accessed on 6 April 2021).

In Egypt, no study was conducted on vaccinated people to detect the post Sinopharm vaccine symptoms and assess its efficacy. The study survey was designed based on the information published on the website of the FDA [12].

The survey items are inclusive as they include post-Sinopharm vaccine first dose symptoms and practices, post-Sinopharm vaccine second dose symptoms and practices, and the results of IgG anti spike protein antibodies tests and laboratory tests at day 18 after vaccination [12].

A lot of the participants were younger in age and were females. They were more interested in participating in the survey and sharing their experience than other groups. That may be due to the fact that young people and women suffer more from post-vaccine adverse effects than elderly people and men. Previous research [13,14] found that older people have fewer post-vaccine side effects and a lower immune response than younger people A recent study showed that women experience more side effects than men and noted that this could be related to men having higher testosterone levels, which may contribute to men experiencing fewer side effects [14].

Health care workers have a positive attitude towards vaccination due to their constant contact with COVID-19 patients to protect themselves from the risk of infection shown in the study by Rahul Shekhar et al. [15,16]. The majority of the participants suffered from post-coronavirus vaccine syndrome. Pain, redness, or swelling where the vaccine was injected, fatigue, and lethargy were the most common post coronavirus vaccine syndrome for both the first and second doses [15,17,18].

About 10% of the participants received the second dose after more than four weeks of receiving the first dose, as they were infected with COVID-19 after receiving the first dose. This may be related to the small immune response induced after the first dose of vaccination [10].

The post-vaccine symptoms after the first and second doses are commonly mild, as classified by the participants. The symptoms were mild, not serious, and did not threaten life [11,18]. Similar to the findings of an earlier study, it is noticeable that a higher percentage of participants did not feel any side effects after the second dose of vaccination than after the first dose. [18] However, for the participants who suffered from post-vaccine symptoms, the severity of the symptoms was greater after the second dose than after the first dose. The Centers for Disease Control and Prevention reported that side effects after the second dose are more intense [19]. Most of the participants had post-vaccine symptoms after the first dose and persisted for two days or more, which may be attributed to the immune system response [18]. The cytokines produced by the immune system have inflammatory effects on the muscles, blood vessels, and other body organs. Therefore, the post-coronavirus vaccine symptoms lasted for days after receiving the vaccine [20].

A small percentage of the participants took pain relievers after taking the second dose of the vaccine. After vaccination, the protective immune response is mainly dependent on the sufficient production of antibodies. Some people use non-steroidal anti-inflammatory drugs (NSAIDs) to treat post-coronavirus vaccine symptoms. Some studies reported concerns about using NSAIDs for treating symptoms of coronavirus or post coronavirus vaccine symptoms [11,21]. NSAIDs inhibit cyclooxygenase 1 (COX 1) and cyclooxygenase 2 (COX 2) enzymes and inflammatory cytokines. Cox enzymes are necessary for the production of sufficient antibodies in response to coronavirus vaccine or infection [11,21]. Therefore, the use of NSAIDs may reduce the production of antibodies in response to coronavirus vaccination or infection [11,21].

A previous study showed that the most-reported adverse effects for the Oxford/AstraZeneca COVID-19 vaccine were injection site pain and tenderness, tiredness, and headache. The side effects started on the first day after the vaccination and lasted for 1–3 days. Severe side effects were uncommon, but they were a major reason why vaccinated people did not recommend it to others and did not accept receiving the second dose [10].

The most common side effects for Pfizer/BioNTech were pain at the injection site, headaches, fever, flu-like symptoms, and tiredness [14]. The side effects started on the first day after the vaccination and lasted for 1–3 days.

Although post-vaccination side effects are the same for all vaccines, the number and severity of these side effects significantly differ according to the vaccine type (Oxford/AstraZeneca > Pfizer/BioNTech > Sinopharm) [22].

The average of the laboratory factors of the participants showed normal results except for the Erythrocyte Sedimentation Rate (ESR). ESR relatively increased in participants after vaccination. Increased ESR may be due to inflammation or false infection. That was also reported in a previous study [14]. About 16% of the participants were infected with coronavirus during the last four months before vaccination. The level of antibodies after infection is considered to drop over months, so the participants infected during the last four months before vaccination probably still have post-infection antibodies that could appear in the post-vaccine antibodies test.

Slightly more than half of the participants showed negative results for the IgG anti-spike protein antibodies test on day 18 after vaccination. This high percentage of negative results for the antibodies test indicates that the Sinopharm vaccine cannot produce a sufficient immune response in most vaccinated cases. Researchers in Brazil found 50.7% efficacy for the Sinovac vaccine, or 62.3% with longer dosing intervals [23], while researchers in Turkey found 83.5% efficacy [24]. Indonesia reported 65% efficacy [25].

About 49% of the participants resulted in a positive IgG anti spike protein antibody test, involving 16% of the participants who had a past coronavirus infection. The positive immune response seen in the vaccine antibody test results for 16% of previously infected participants could be attributed to the previous infection rather than the vaccination. Then the confirmed positive antibody test resulted from Sinopharm vaccines appeared in only 33% of the vaccinated participants. The level of antibodies was much lower in the 33% of non-past infected vaccinated participants with positive antibodies tests compared to the 16% of past infected vaccinated participants. This indicates that the Sinopharm vaccine cannot trigger sufficient antibodies as an immune response in most vaccinated cases. The elderly were more likely to have negative antibody test results. A previous study suggested that post-vaccine symptoms were more common among younger individuals and reported that the symptoms are related to the immune response associated with vaccines and the antibodies produced [15].

The average of positive results of the quantitative IgG anti-spike protein antibodies test for cases with a previous coronavirus infection during the four months before vaccination was much higher than those with no past infection. That may be because the vaccine is like a second infection, so people vaccinated after an infection develop a better response to the vaccine. The bodies of vaccinated cases with past coronavirus infection respond better than those who are only vaccinated, as the antibody concentrations are usually much higher in vaccinated and infected people than in people who are only vaccinated or only infected. In addition, a previous study reported that one dose of coronavirus vaccine is sufficient in subjects infected with coronavirus before reaching a satisfactory level of antibodies. They suggested this idea to overcome the current shortage of vaccines [23].

The head of China’s Centre for Disease Prevention and Control said that the Chinese vaccines (Sinopharm) need to be replaced by more effective vaccines or changed in their administration to make them satisfactory effective against the coronavirus infection [16]. These vaccines have low protection rates, so different vaccines with different techniques should be used for more protection. The efficacy problem of these Chinese vaccines may be solved by changing the dose, dose interval, increasing the number of doses, or mixed with other vaccines using different techniques. The United Arab Emirates, which uses the Sinopharm vaccine, has recently used it with a third dose of the vaccine to improve the antibody response. The global coronavirus vaccine production shortage is also a big problem, as is its low efficacy [23].

## 5. Conclusions

Pain, redness, or swelling at the injection site, headache, fatigue, and lethargy were the most common post-vaccine symptoms for both the first and second doses. More than half of the participants showed negative results for the IgG anti-spike protein antibodies test after vaccination. The elderly were more likely to have negative antibody test results. The cases with no past coronavirus infection who showed positive results for the IgG anti spike protein antibodies test showed a low level of antibodies in the participants’ blood against coronavirus compared to those with past coronavirus infection during the last four months before vaccination. These vaccines need to be reevaluated in their methods of administration by changing the dose, dose interval, adding a third dose, or mixing it with other vaccines with different techniques to increase their induced immunity and protection rates.

## Figures and Tables

**Table 1:** Shows the demographic data percentages of the participants.

Characteristic	Number (%)
Gender	
Male	18 (15%)
Female	102 (85%)
Age in years	
From 18 to 35 years	57 (47.5%)
From 35 to 45 years	33 (27.5%)
From 45 to 55 years	9 (7.5%)
More than 55 years	21 (17.5%)
Marital status	
Unmarried	31 (26%)
Married	89 (74%)
Current residence place	
City	92 (77%)
Country	28 (23%)
Job	
No work	30 (25%)
I am a health care worker	63 (55%)
I work in another field	24 (20%)

**Table 2:** Symptoms and practices following the first dose of the Sinopharm vaccine.

Question	Number (%)
1—What are the symptoms that you felt after you received the first dose of Sinopharm vaccine?	
• Pain, redness, or swelling where the vaccine was injected	63 (52.5%)
• Fever	12 (10%)
• Sore throat	3 (2.5%)
• Headache	18 (15%)
• Muscle pain	12 (10%)
• Excessive sweating	0%
• Joint pain	9 (7.5%)
• Feeling dizzy	6 (5%)
• Abdominal pain	0%
• Decreased appetite	3 (2.5%)
• Nausea and vomiting	0%
• Diarrhea	0%
• Cough	3 (2.5%)
• Allergies and rashes	3 (2.5%)
• Runny nose	12 (10%)
• Fatigue and lethargy	60 (50%)
• Convulsions	0%
• Swollen lymph nodes	0%
• Any blood clots	0%
• Inflammation of the nervous system, including numbness, tingling and loss of sensation	3 (2.5%)
• I didn’t feel any side effects	30 (25%)
2—To what extent do you rate the severity of these symptoms and complications?	
• Mild	59 (49%)
• Moderate	22 (18%)
• Sever	9 (8%)
• I didn’t feel any side effects	30 (25%)
3—Symptoms appeared after the first dose during	
• The first day after receiving the vaccine	81 (67.5%)
• The second day after receiving the vaccine	3 (2.5%)
• The third day after receiving the vaccine	6 (5%)
• After the third day from receiving the vaccine	0%
• I didn’t feel any side effects	30 (25%)
4—The symptoms that appeared after the first dose persisted for;	
• One day	27 (22.5%)
• Two days	36 (30%)
• More than two days	27 (22.5%)
• I didn’t feel any side effects	30 (25%)
5—You have taken pain relievers.	
• Before taking the first dose of the vaccine	0%
• After taking the first dose of the vaccine	27 (22.5%)
• I didn’t take any painkillers	93 (77.5%)

**Table 3:** Symptoms and practices following the second dose of the Sinopharm vaccine.

Question	Number (%)
1—I took the second dose of the vaccine after	
• Two weeks after the first dose	0%
• Three weeks after the first dose	108 (90%)
• Four weeks after the first dose	0%
• More than four weeks after receiving the first dose	12 (10%)
2—Were you infected with the corona virus between the first and second doses (or you had severe symptoms of corona for more than two days after receiving the first dose)	
• Yes	12 (10%)
• No	108 (90%)
3—What are the symptoms that you felt after you received the second dose of Sinopharm vaccine?	
• Pain, redness, or swelling where the vaccine was injected	15 (12.5%)
• Fever	3 (2.5%)
• Sore throat	6 (5%)
• Headache	12 (10%)
• Muscle pain	15 (12.5%)
• Excessive sweating	0%
• Joint pain	6 (5%)
• Feeling dizzy	3 (2.5%)
• Abdominal pain	0%
• Decreased appetite	0%
• Nausea and vomiting	0%
• Diarrhea	0%
• Cough	0%
• Allergies and rashes	6 (5%)
• Runny nose	9 (7.5%)
• Fatigue and lethargy	54 (45%)
• Convulsions	0%
• Swollen lymph nodes	0%
• Any blood clots	0%
• Inflammation of the nervous system, including numbness, tingling, and loss of sensation	0%
• I didn’t feel any side effects	45 (37.5%)
4—To what extent do you rate the severity of these symptoms and complications?	
• Mild	39 (32.5%)
• Moderate	27 (22.5%)
• Sever	9 (7.5%)
• I didn’t feel any side effects	45 (37.5%)
5—Symptoms appeared after the second dose during	
• The first day after receiving the vaccine	54 (45%)
• The second day after receiving the vaccine	15 (12.5%)
• The third day after receiving the vaccine	0%
• After the third day from receiving the vaccine	6 (5%)
• I didn’t feel any side effects	45 (37.5%)
6—The symptoms that appeared after the second dose persisted for	
• One day	27 (22.5%)
• Two days	18 (15%)
• More than two days	30 (25%)
• I didn’t feel any side effects	45 (37.5%)
7—You have taken pain relievers	
• Before taking the second dose of the vaccine	0%
• After taking the second dose of the vaccine	24 (20%)
• I didn’t take any painkillers after taking the second dose of the vaccine.	96 (80%)

**Table 4:** The results of IgG anti spike protein antibodies test and laboratory tests at day 18 after vaccination with Sinopharm vaccines.

Question	Results
1—Have you had corona infection before?	
• Yes	16%
• No	84%
2—Results of IgG anti spike protein antibodies test	
• Positive (more than 1)	49%
• Negative (less than 1)	51%
3—Average of positive results of quantitative IgG anti spike protein antibodies test for the cases with a previous coronavirus infection during the four months before vaccination (for 16% of the participants)	147 AU/mL
4—Average of positive results of quantitative IgG anti spike protein antibodies test for the cases with no previous coronavirus infection before vaccination (for 33% of the participants)	40 AU/mL
5—Gender	
Positive positive results of quantitative IgG anti spike protein antibodies test	
• Male	52%
• Female	48%
Negative positive results of quantitative IgG anti spike protein antibodies test	
• Male	24%
• Female	76%
Average of positive results of quantitative IgG anti spike protein antibodies test	
• Male	83 AU/mL
• Female	50 AU/mL
6—Age in years	
Positive positive results of quantitative IgG anti spike protein antibodies test	
• From 18 to 40 years	75%
• From 41 to 60 years	20.80%
• More than 60 years	4.20%
Negative positive results of quantitative IgG anti spike protein antibodies test	
• From 18 to 40 years	45%
• From 41 to 60 years	33%
• More than 60 years	22%
Average of positive results of quantitative IgG anti spike protein antibodies test	
• From 18 to 40 years	72 AU/mL
• From 41 to 60 years	56 AU/mL
• More than 60 years	29 AU/mL
7—Average of laboratory parameters for the positive IgG anti spike protein antibodies cases	
- Hemoglobin	12.6
- Platelets	263
- Leucocytes	7.9
- Neutrophils	53%
- Lymphocytes	42%
- ESR	21
- SGOT	20
- SGPT	20.5

## Data Availability

Data are available upon request.
